# Sustained viral response and relapse after discontinuation of oral antiviral drugs in HBeAg-positive patients with chronic hepatitis B infection

**DOI:** 10.3389/fimmu.2022.1082091

**Published:** 2022-11-25

**Authors:** Fangfang Sun, Zhenhua Li, Leiping Hu, Wen Deng, Tingting Jiang, Shiyu Wang, Xiaoyue Bi, Huihui Lu, Liu Yang, Yanjie Lin, Zhan Zeng, Ge Shen, Ruyu Liu, Min Chang, Shuling Wu, Yuanjiao Gao, Hongxiao Hao, Mengjiao Xu, Xiaoxue Chen, Lu Zhang, Yao Lu, Jianping Dong, Yao Xie, Minghui Li

**Affiliations:** ^1^ Department of Hepatology Division 2, Beijing Ditan Hospital, Capital Medical University, Beijing, China; ^2^ Department of Infectious Diseases, Haidian Hospital, Beijing Haidian Section of Peking University Third Hospital, Beijing, China; ^3^ Department of Gynecology and Obstetrics, Beijing Ditan Hospital, Capital Medical University, Beijing, China; ^4^ Department of Obstetrics and Gynecology, Wuhan Children’s Hospital (Wuhan Maternal and Child Healthcare Hospital), Tongji Medical College, Huazhong University of Science and Technology, Wuhan, China; ^5^ Department of Hepatology Division 2, Peking University Ditan Teaching Hospital, Beijing, China

**Keywords:** chronic hepatitis B, HBeAg, HBV DNA, nucleos(t)ide analogue, clinical relapse, sustained virological response

## Abstract

**Objective:**

To investigate the sustained virological response and relapse in chronic hepatitis B (CHB) patients with hepatitis B e antigen (HBeAg) positive after stopping oral antiviral drugs, and to monitor the disease progression and the incidence of adverse events such as liver cirrhosis and hepatocellular carcinoma.

**Methods:**

This is a prospective observational study. Patients who continued nucleos(t)ide analogue (NA) treatment after achieving HBeAg seroconversion for more than 3 years were enrolled. After signing the informed consent form, patients stopped NA treatment and received follow-up. During the follow-up, the antiviral treatment information of the patients was collected, and the follow-up observation was carried out every 3 months since the enrollment. We monitored the virological indexes, liver and kidney function, serology and liver imaging during follow-up. The purpose of this study was to explore the sustained virological response rate, HBV DNA recurrence rate, clinical relapse rate and the related factors after drug withdrawal.

**Results:**

A total of 82 patients were enrolled, including 42 males (51.22%) and 40 females (48.78%), with a median age of 34.00 (31.00, 37.25) years. All enrolled patients were followed up for 1 year. At the end of the follow-up, 36.59% (30/82) of patients had sustained virological response, 63.41% (52/82) of patients had HBV DNA reactivation, 17.07% (14/82) of patients had clinical relapse, and 10.98% (9/82) of patients had HBeAg reversion. During the follow-up, there were no adverse events such as liver cirrhosis and hepatocellular carcinoma. The median level of hepatitis B surface antigen (HBsAg) in patients with sustained virological response was lower than that in patients with HBV DNA reactivation (2.92 vs.3.18 log_10_IU/ml, *Z*=-1.492/*P*=0.136), and the median level of baseline HBsAg in patients with HBV DNA reactivation was lower than that in patients with clinical relapse (3.01 vs.3.45 log_10_IU/mL, *Z*=-1.795/*P*=0.073), but the difference was not significant. There was no significant statistical difference between patients with sustained virological response and HBV DNA reactivation of the median total treatment time [69.50 (56.25, 86.00) vs.62.50 (44.00, 88.50) months, *Z*=-0.689/*P*=0.491], and the consolidation treatment time [41.50 (36.75, 54.75) vs.40.50 (36.00, 53.75) months, *Z*=-0.419/*P*=0.675].

**Conclusion:**

The sustained virological response rate of HBeAg positive CHB patients after stopping oral antiviral treatment is lower, and it is more common in patients with lower HBsAg levels. Patients still need to be closely monitored after stopping NA therapy.

## Introduction

CHB is a world public health problem. According to the statistics of the World Health Organization, 296 million people were living with chronic hepatitis B virus (HBV) infection in 2019. CHB resulted in an about 820000 deaths, mainly due to cirrhosis and hepatocellular carcinoma (1, 2). CHB is an immune-mediated liver inflammatory injury. In the pathogenesis of CHB, the virus stimulates the host to cause immune response, and then immune factors induce the inflammation of liver tissue and liver cell damage (3–6). It is generally believed that patients can inhibit virus replication through antiviral treatment, thereby reducing the stimulation of virus on immunity, alleviating liver inflammation, reducing the occurrence of cirrhosis and hepatocellular carcinoma, prolonging the life span of patients and improving the quality of life (3, 4, 7, 8).

NA is a commonly used oral antiviral drug, which is widely used because of its strong antiviral effect, convenient administration and a few side effects. However, patients often need long-term medication, for NA cannot improve the host’s immune control of the virus. Long term drug use will also bring many problems: although there are few adverse reactions of NA, its long-term safety remains to be confirmed (9). Long term treatment has significantly increased the economic burden of the national health care system. In many regions, the cost of NA treatment cannot be reimbursed or can only be partially reimbursed, bringing great economic burden to individuals and families. It is difficult to define the time of drug withdrawal which brings out great psychological pressure to patients. What needs more attention is that even if the drug is given for a long time, it is difficult to induce a lasting response after drug withdrawal. It is unclear how long NA should be treated, which drug withdrawal criteria is most effective and conforms to the principles of health economics, and which indicators can effectively predict the relapse after drug withdrawal. Therefore, it is particularly important to formulate a reasonable and feasible study to stop oral antiviral treatment.

In recent years, some scholars have proposed that CHB patients receiving long-term oral antiviral treatment may benefit from drug withdrawal. Liver inflammatory mediated by immune response after drug withdrawal is more likely to induce virus clearance and achieve negative transformation of HBsAg (10–13). In 2015, China’s prevention and treatment guidelines for CHB proposed that drug withdrawal can be considered when patients with HBeAg positive achieved HBeAg serological conversion, HBV DNA turns negative and continued to consolidate treatment for 3 years (14). However, whether patients can maintain virological response after stopping treatment and the safety after stopping treatment still need our further research to verify.

In this study, patients with CHB who met the drug withdrawal criteria were followed up after drug withdrawal, to determine the sustained virological response rate of patients after stopping oral antiviral treatment and explore its related influencing factors, in order to provide help for us to explore the drug withdrawal mode of HBeAg positive CHB patients.

## Materials and methods

### Subjects and study design

This is a prospective study of CHB patients who were admitted to the Department of Hepatology Division 2 of Beijing Ditan Hospital from January 1, 2017 to December 30, 2020. Eligible patients who were HBeAg positive before treatment and stopped taking drugs in accordance with the Chinese Guidelines for the Prevention and Treatment of Chronic Hepatitis B (2015) were rolled. This study was approved by the Ethics Committee of Beijing Ditan Hospital affiliated to Capital Medical University (Jing Di Lun Ke Zi 2017 No. 003 - 02).

Inclusion criteria: 1) Age above 18 (including 18 when signing the informed consent form); 2) Gender is not limited; 3) HBsAg (i.e. HBsAg ≥ 0.05 IU/mL) is positive for more than 6 months; 4) HBeAg positive (HBeAg ≥ 1 S/CO) before antiviral treatment; 5) Use oral antiviral drugs such as nucleus (t) ide analog (NA) for treatment; 6) Meet the drug withdrawal standard of the Chinese Guidelines for the Prevention and Treatment of Chronic Hepatitis B (2015), and have continued treatment for at least 3 years after HBV DNA negative conversion and HBeAg seroconversion (14); 7) Volunteer to participate in this study, sign the informed consent form, and be able to participate in the follow-up on schedule.

Exclusion criteria: 1) Those who have used interferon antiviral therapy before; 2) The patients were treated with glucocorticoid, thymosin and other immunomodulators before and during the follow-up period; 3) Patients with liver cirrhosis or liver cancer; 4) During screening, alpha fetoprotein (AFP) and liver imaging indicated that patients may have malignance; 5) Patients with other virus infections (HCV, HDV, HIV, EBV, CMV, etc); 6) Complicated with autoimmune liver disease, alcoholic liver disease, genetic metabolic liver disease, fatty liver disease, drug induced liver disease and other liver diseases; 7) Patients with other malignant tumors or undergoing immunomodulation therapy due to other diseases; 8) The investigator believes that patients who are not suitable to participate in this study.

Follow-up time: Follow up every 3 months from the cessation of antiviral treatment to 1 year after the cessation of antiviral treatment.

### Collection of clinical data

1) Demographic data: patient’s sex, age, family history of liver cancer, transmission route;
2) Collection of enrollment data: antiviral drug treatment information, including treatment drugs, time of starting antiviral treatment, total antiviral treatment time, consolidation treatment time, application history of other drugs, biochemical indicators before and during treatment, virological indicators, immunological indicators, imaging data, etc., and complications before and during treatment. However, we failed to collect HBV genotype at the enrollment.
3) Monitoring indicators during the follow-up: patients were monitored every 3 months during the follow-up, including: alanine aminotransferase (ALT), aspartate aminotransferase (AST), direct bilirubin (DBIL), total bilirubin (TBIL), albumin (ALB), AFP, blood routine, HBsAg, HBeAg, HBeAb, HBV DNA, abdominal ultrasound, liver elasticity examination, etc.

### Biochemical examination

HBV serological markers were measured by chemiluminescent microparticle immunoassay (Architect i2000 analyzer; Abbott Laboratories). The detection range of HBsAg was 0.05-250 IU/mL, HBeAg ≥ 1 S/CO was positive, and HBeAb < 1 was positive. Serum HBV DNA was detected by fluorescence quantitative polymerase chain reaction (Roche, Switzerland, Light Cycle 480 PCR system), and the lower limit of detection value was 20 IU/mL. The liver function index was detected with Hitachi 7600 automatic biochemical analyzer, and the upper limit of normal ALT value was 40U/L (Osaka and TanPUR Chemical Industry Co., Ltd., Japan).

### Off-therapy outcome measures

Main evaluation measures: The sustained off-treatment virological response rate after discontinuation of oral antiviral therapy, which was defined as HBV DNA remained below the lower limit of detection after drug withdrawal during the follow-up.

Secondary evaluation indexes: 1) Virological re-positive rate; 2) Clinical relapse rate; 3) HBeAg reversion rate; 4) HBsAg negative rate; 5) Incidence rate of adverse events such as liver cirrhosis and liver cancer.

### Various definitions

1) Antiviral treatment time: the time from the start of antiviral treatment to drug withdrawal;
2) Consolidation treatment time: The time from HBeAg serological conversion and HBV DNA negative conversion to stop antiviral treatment for HBeAg positive patients during the treatment period;
3) Sustained off-treatment virological response: HBV DNA level of CHB patients is still lower than the lower limit of detection after stopping oral antiviral treatment 1 year;
4) Viral relapse: HBV DNA were positive for two consecutive times (3 months apart) after stopping oral antiviral treatment;
5) Clinical relapse: Patients’ HBV DNA were positive and ALT ≥ 2×ULN at the same visit (the upper limit of ALT detection value in our hospital is 40U/L);
6) HBeAg reversion: Patients’ HBeAg appear positive again after HBeAg seroconversion;
7) HBsAg negative: Patients with chronic HBV infection show sustained off-treatment viral response, and the level of HBsAg is less than 0.05 IU/mL;
8) Liver fibrosis score (APRI, FIB-4 score): APRI ≥ 2 indicates liver cirrhosis, APRI < 1 excludes liver cirrhosis (3), FIB-4 ≥ 3.25 indicates progressive fibrosis, FIB-4 < 1.45 excludes progressive liver fibrosis (15, 16).

### Statistical analysis

The continuous variables were presented as mean, standard deviation, median and interquartile range. T-tests and Mann-whitney U test were used for comparison between two groups. The categorical variables were presented as frequencies and proportions. The chi-square analysis and Fisher test were used to compare between the two groups. Pearson and Spearman correlation analysis were used for correlation analysis of two continuous variables. Logistic regression was used to analyze the influencing factors of sustained off-treatment virological response. All tests were performed on both sides, and P<0.05 was defined as statistically significant. Biochemical, viral and serological indicators were observed at five time points and *P*<0.01 was statistically significant. For all analyses, SPSS software (version 19.0) was used.

## Result

### Baseline demographic and clinical characteristics of enrolled patients

**Figure 1:**
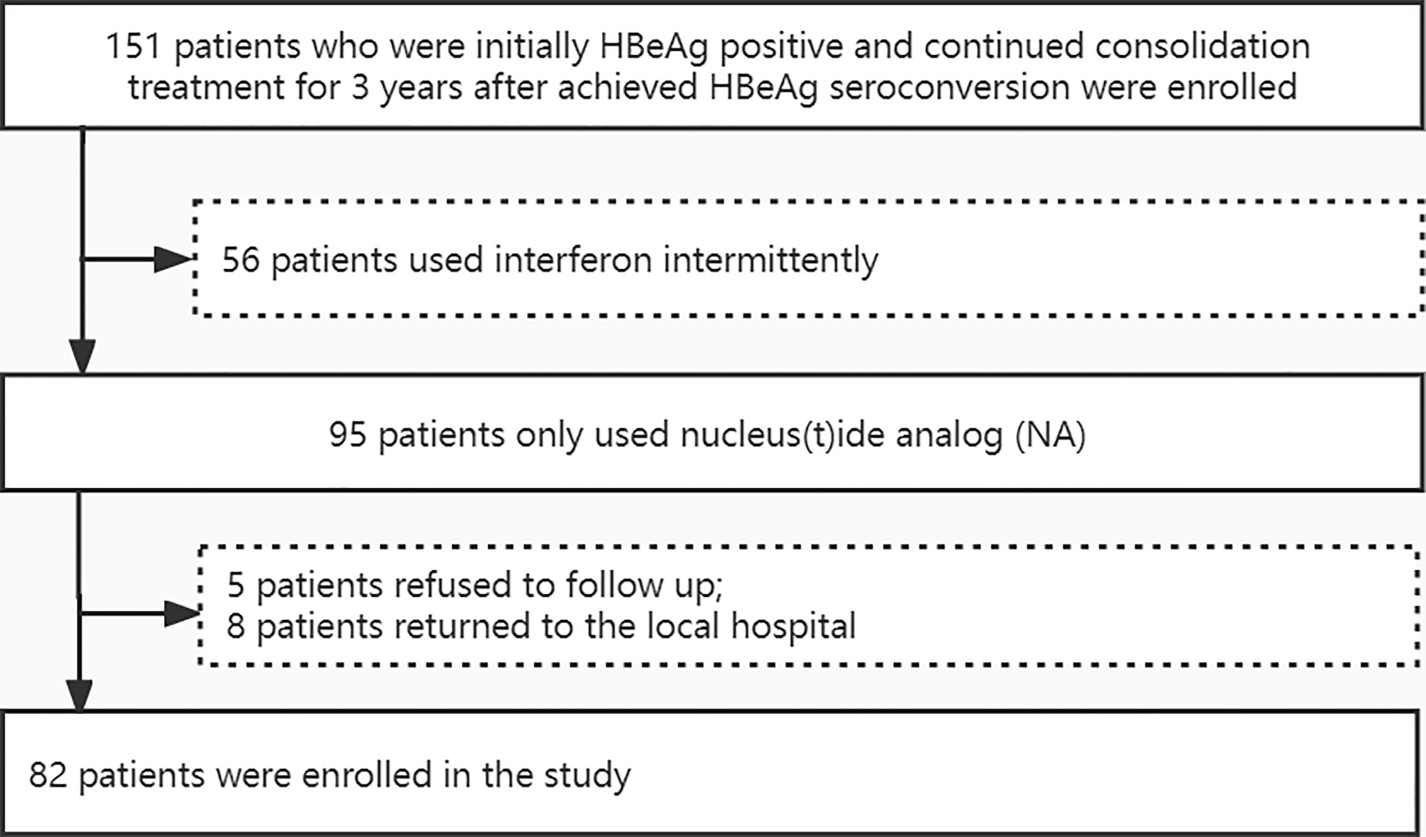
Patient enrollment and deposition.

A total of 151 patients were initially HBeAg positive and continued consolidation treatment for 3 years after achieved HBeAg seroconversion. Among them, 56 patients used interferon intermittently, 5 patients refused to follow up, and 8 patients could not come to the hospital regularly because they returned to the local hospital. Finally, 82 patients were included in this study (**Figure 1**).

Of the 82 patients, 42 were male (51.22%) and 40 were female (48.78%), with a median age of 34.00 (31.00, 37.25) years old. Eight patients (9.76%) had a family history of liver cancer. Among the infection routes of all enrolled patients, 31 (37.80%) patients were infected with HBV by mother-to-child transmission, 24 (29.27%) patients had a long-term contact history with hepatitis B patients, 2 (2.44%) patients may be infected through infusion or surgery, 1 (1.22%) patient had a history of blood transfusion and close contact with hepatitis B patients, and the others were unknown.

The median total treatment time was 66.50 (52.75, 86.25) months, and the median consolidation treatment time was 41.00 (36.00, 54.00) months. During the treatment, some patients changed the oral antiviral drug due to drug resistance, drug safety and other side effects. 46 patients (56.10%) used only one oral antiviral drug, 27 patients (32.93%) used two oral antiviral drugs, and 9 patients (10.98%) used three oral antiviral drugs successively. Before stopping antiviral treatment, 62 patients (75.61%) used entecavir (ETV), and 17 patients (20.73%) used tenofovir disoproxil fumarate (TDF), two patients (2.44%) used adefovir dipivoxil (ADV) and one patient (1.22%) used lamivudine (LAM) antiviral therapy.

The median level of HBsAg was 3.06 (2.79, 3.51) log_10_ IU/mL when patients stopped oral antiviral therapy, of which 12.20% (10/82) patients had HBsAg ≤ 100 IU/mL, and 87.80% (72/82) patients had HBsAg > 100 IU/mL. All patients had normal liver function, and the median APRI score at baseline was 0.26 (0.19, 0.44). According to APRI score, no patient had cirrhosis, and 79 patients could completely exclude cirrhosis. The median FIB-4 score at baseline was 0.85 (0.63, 1.22). According to the FIB-4 score, no patients considered advanced liver fibrosis, and 72 patients could exclude advanced liver fibrosis. No patients had liver cirrhosis or malignant tumor according to their imaging examination (**Table 1**).

**Table 1:** Baseline characteristic of the subjects.

Clinical features	Values
Age [year, median (Q1, Q3)]	34.00 (31.00, 37.25)
Male (n, %)	42 (51.22%)
Family history of liver cancer (n, %)	8 (9.76%)
Antiviral treatment time [month, median (Q1, Q3)]	66.50 (52.75, 86.25)
Consolidation treatment time [month, median (Q1, Q3)]	41.00 (36.00, 54.00)
Kinds of oral antiviral treatment drugs (n, %)	
1	46 (56.10%)
2	27 (32.93%)
3	9 (10.98%)
HBsAg (log_10_ IU/mL)	3.06 (2.79, 3.51)
HBsAg (IU/mL)	
≤100	10 (12.20%)
>100	72 (87.80%)
AFP (ng/mL)	2.20 (1.70, 3.23)
ALT (U/L)	19.65 (12.78, 33.05)
AST (U/L)	20.15 (17.48, 26.25)
TBIL (umol/L)	10.50 (8.38, 13.63)
DBIL (umol/L)	3.80 (3.10, 4.90)
ALB (g/L)	47.50 (45.03, 49.23)
APRI [median (Q1, Q3)]	0.26 (0.19, 0.44)
APRI≥2 (n, %)	0
APRI<1 (n, %)	79 (96.34%)
FIB-4 [median (Q1, Q3)]	0.85 (0.63, 1.22)
FIB-4≥3.25 (n, %)	0
FIB-4<1.45 (n, %)	72 (87.80%)

82 patients were enrolled in the study; Values are presented as n (%), or median (Q1, Q3).

HBsAg, hepatitis B surface antigen; AFP, alpha fetoprotein; ALT, alanine aminotransferase; AST, aspartate aminotransferase; TBIL, total bilirubin; DBIL, direct bilirubin; ALB, albumin;

APRI = [AST (U/L)/ULN ×100]/PLT (10^9^/L) note: ULN= the upper limit of AST (40U/L)

FIB-4 = [Age ×AST (U/L)]/[PLT (10^9^/L) ×√ALT (U/L)]

APRI≥2 represents liver cirrhosis, APRI<1 excludes liver cirrhosis; FIB-4 ≥3.25 represents progressive liver fibrosis, FIB-4<1.45 excludes progressive liver fibrosis.

### Different prognosis after discontinuation of oral antiviral therapy

**Figure 2:**
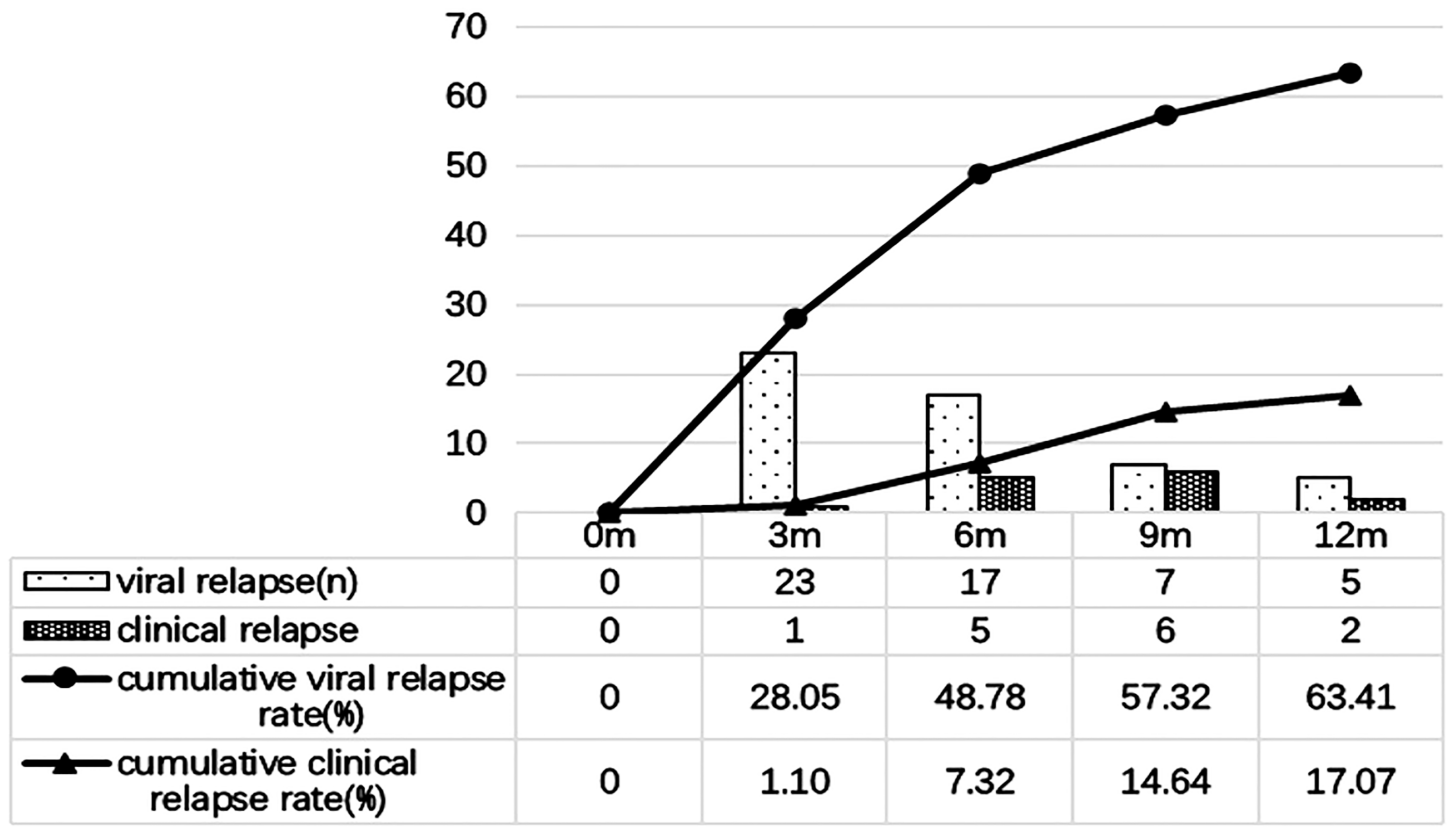
Viral relapse and clinical relapse in patients after stopping oral antiviral treatment.

Within 12 months after discontinuing oral antiviral therapy, 36.59% (30/82) of the patients maintained virological response, 63.41% (52/82) of the patients had virological relapse, of which 28.05% (23/82) relapse at 3month, 20.73% (17/82) relapse at 6month, 8.54% (7/82) relapse at 9 month, and 6.10% (5/82) relapse at 12month. 17.07% (14/82) of patients had clinical relapse during the follow-up, of which 1.22% (1/82) had clinical relapse at 3 month, 6.10% (5/82) had clinical relapse at 6 month, 7.32% (6/82) had clinical relapse at 9 month, and 2.44% (2/82) had clinical relapse at 12 month (**Figure 2**).

In this study, 10.98% (9/82) of patients had HBeAg reversion during the 12-month follow-up period, which all occurred in patients with clinical relapse, and mainly occurred at 6 months after stopping treatment. During the follow-up, 3.66% (3/82) of the patients had HBsAg decrease > 1 log_10_ IU/mL, no patients achieved HBsAg negative, and no patients developed to cirrhosis and liver cancer.

Clinical relapse means that HBV replication in patients is active, which induces the immune defense of the body and causes hepatitis. Some patients may progress to liver failure during this process. Therefore, all patients with clinical relapse received antiviral treatment again for safety in our study, of which 7 patients (50.00%) received ETV 0.5 mg daily, 3 patients (21.43%) received TDF 300 mg daily, and 1 patient (7.14%) received pegylated interferon α- 2a 180ug combined with ETV 0.5 mg, 2 patients (14.29%) received pegylated interferon α- 2a 180ug combined with TDF 300mg, one patient (7.14%) received pegylated interferon α- 2a 180ug combined with 25 mg propofol tenofovir fumarate. The patients treated with pegylated interferon α- 2a and ETV achieved clinical cure, and the remaining patients were negative for HBV DNA after treatment, and the patients with HBeAg reversion achieved HBeAg serological conversion again.

### Characteristic of patients with sustained virological response and viral relapse at baseline

In this study, 30 (36.59%) patients had sustained virological response and 52 (63.41%) patients had viral relapse after 12-months of follow-up. The comparison of the characteristic between the two groups showed that the median age of patients in the sustained virological response group [34.50 (30.75, 39.25) vs.33.50 (31.00, 37.00) years, *Z*=-0.391/*P*=0.696], antiviral treatment time [69.50 (56.25, 86.00) vs.62.50 (44.00, 88.50) months, *Z*=-0.689/*P*=0.491], the consolidation treatment time [41.50 (36.75, 54.75) vs 40.50 (36.00, 53.75) months, *Z*=-0.419/*P*=0.675], had no significant difference with viral relapse patients. There was no significant difference between the two groups in gender, family history of liver cancer and the kinds of oral antiviral drugs (*P*>0.05). The median level of HBsAg in the sustained virological response group was lower than that in the viral relapse group, but the difference was not statistically significant [2.92 (2.36, 3.52) vs.3.18 (2.90, 3.54) log_10_ IU/mL, *Z*=-1.492/*P*=0.136]. The ALT, AST, TBIL, DBIL, ALB and other indicators of the patients in the sustained virological response group and the viral relapse group were not significantly different. The baseline median APRI of the sustained virological response group was 0.28 (0.18, 0.41), and that of the viral relapse group was 0.25 (0.21, 0.33). According to the APRI score, 29 (96.70%) patients in the sustained virological response group could exclude cirrhosis, and 50 (96.20%) patients in the viral relapse group could exclude cirrhosis. In addition, the median baseline FIB-4 in the sustained virological response group was 0.61 (0.49, 1.05), and that in the viral relapse was 0.74 (0.58, 0.90). According to the FIB-4 score, 26 (86.70%) patients in the sustained virological response group could exclude advanced liver fibrosis, and 46 (88.50%) patients in the viral relapse group could exclude advanced liver fibrosis (**Table 2**).

**Table 2:** Characteristic of patients with sustained virological response and Viral relapse at baseline.

	Sustained virological response (n=30)	Viral relapse (n=52)	*Z/χ^2^*	*P*
Age [year, median (Q1, Q3)]	34.50 (30.75, 39.25)	33.50 (31.00, 37.00)	-0.391	0.696
Male (n, %)	14 (46.70%)	28 (53.80%)	0.392	0.531
Family history of liver cancer (n, %)	2 (8.70%)	6 (11.50%)	0.109	0.742
Kinds of oral antiviral treatment drugs (n, %)				0.944
1	18 (60.00%)	28 (53.80%)		
2	9 (30.00%)	18 (34.60%)		
3	3 (10.00%)	6 (11.50%)		
Antiviral treatment time [month, median (Q1, Q3)]	69.50 (56.25, 86.00)	62.50 (44.00, 88.50)	-0.689	0.491
Consolidation treatment time [month, median (Q1, Q3)]	41.50 (36.75, 54.75)	40.50 (36.00, 53.75)	-0.419	0.675
HBsAg (log_10_ IU/mL)	2.92 (2.36, 3.52)	3.18 (2.90, 3.54)	-1.492	0.136
HBsAg ≤ 100 IU/mL	6 (20.00%)	4 (7.70%)	1.665	0.197
AFP (ng/mL)	2.00 (1.78, 3.15)	2.20 (1.70, 3.28)	-0.578	0.563
ALT (U/L)	19.05 (12.70, 35.70)	20.45 (12.90, 29.20)	-0.212	0.832
AST (U/L)	21.35 (17.38, 29.03)	19.40 (17.42, 24.93)	-0.361	0.718
TBIL (umol/L)	10.60 (8.28, 13.60)	10.45 (8.40, 14.10)	-0.274	0.784
DBIL (umol/L)	3.80 (3.08, 5.53)	3.75 (3.10, 4.68)	-0.024	0.981
ALB (g/L)	46.50 (44.58, 48.32)	47.80 (45.40, 49.78)	-1.931	0.054
APRI [median (Q1, Q3)]	0.28 (0.18, 0.41)	0.25 (0.21, 0.33)	-0.241	0.810
APRI<1 (n, %)	29 (96.670%)	50 (96.15%)	0	1.000
FIB-4 [median (Q1, Q3)]	0.61 (0.49, 1.05)	0.74 (0.58, 0.90)	-0.289	0.773
FIB-4<1.45 (n, %)	26 (86.67%)	46 (88.46%)	0	1

### Changes in clinical indicators of patients with sustained virological response and viral relapse during follow-up

**Figure 3:**
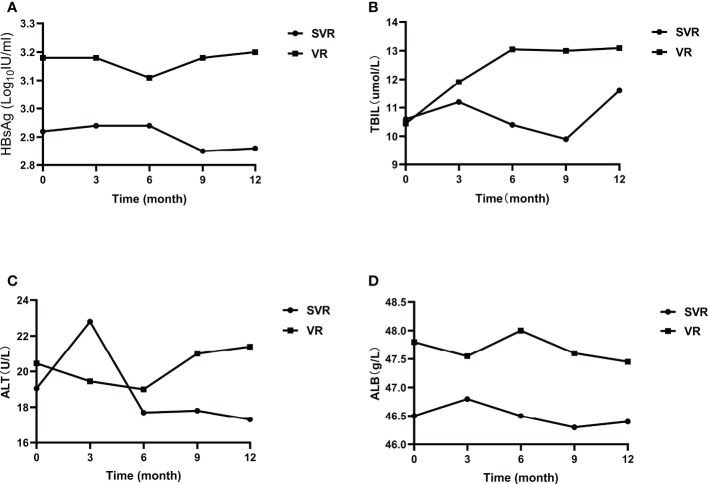
Clinical indicators of patients with sustained virological response and viral relapse. **(A)** the change of HBsAg; **(B)** the change of TBIL; **(C)** the change of ALT; **(D)** the change of ALB. SVR, sustained virological response; VR, viral relapse.

After CHB patients stopped oral antiviral treatment, HBsAg, ALT, TBIL, ALB and other indicators did not change significantly at different time. The median of the above clinical indicators between the two groups were compared (**Figure 3**). The levels of HBsAg in patients with viral relapse at different time were higher than those in sustained virological response patients, but there was no statistically difference (*P*>0.01). The median ALT, TBIL, ALB levels of viral relapse patients at different time were also higher than those in sustained virological response patients, and the differences were not significant (*P*>0.01). In the sustained virological response group, HBsAg level decreased slightly after 6 months of follow-up, while it increased slightly in the viral relapse group. The ALT level of patients with sustained virological response also decreased slightly after 6 months, while that of patients with viral relapse increased slightly.

### Characteristic of patients with clinical relapse and viral relapse at baseline

During the follow-up, there were 52 patients (63.41%) had viral relapse, 14 of whom (26.92%) had clinical relapse. Baseline characteristics were compared between the two groups (**Table 3**). It shows that the median age of viral relapse patients at baseline was 32.50 (30.75, 37.00) years, and the median age of clinical relapse patients was 34.00 (32.00, 40.25) years. There was no significant difference between the two groups (*P*=0.315). In the viral relapse group, the median antiviral treatment time was 60.50 (44.00, 89.50) months, the median consolidation treatment time was 40.50 (36.00, 53.25) months, the median antiviral treatment time for patients with clinical relapse was 65.50 (40.75, 88.25) months, and the median consolidation treatment time was 43.00 (36.00, 56.00) months. There was no significant difference between groups (*P*>0.05). There were no significant differences in sex, family history of liver cancer, and types of antiviral drugs between the two groups (*P*>0.05). The baseline median HBsAg level of viral relapse patients was lower than that of patients with clinical relapse [3.01 (2.87, 3.46) vs.3.45 (3.15, 3.65) log_10_IU/mL, *Z*=-1.795/*P*=0.073]. In this study, 10.53% (4/38) of patients in viral relapse group had HBsAg ≤ 100 IU/mL, and all the patients in clinical relapse group was > 100 IU/mL. However, the difference between the two groups was not significant. The baseline AFP, ALT, TBIL, ALB and other indicators of viral relapse patients and clinical relapse patients were compared, and there was no significant difference between the two groups. According to APRI score, 94.74% (36/38) of viral relapse patients could exclude cirrhosis, and all the patients with clinical relapse could exclude cirrhosis. According to FIB-4 score, 84.21% (32/38) of viral relapse patients could exclude advanced liver fibrosis, and all patients with clinical relapse could exclude advanced liver fibrosis.

**Table 3:** Characteristic of patients with clinical relapse and viral relapse at baseline.

	Viral relapse (n=38)		clinical relapse (n=14)	*Z/χ^2^*	*P*
Age [year, median (Q1, Q3)]	32.50 (30.75, 37.00)	34.00 (32.00, 40.25)		-1.006	0.315
Male (n, %)	22 (57.89%)	6 (42.86%)		0.931	0.335
Family history of liver cancer (n, %)	4 (10.50%)	2 (14.30%)		0	1.000
Kinds of oral antiviral treatment drugs (n, %)					0.490^*^
1	21 (55.26%)	7 (50.00%)			
2	14 (36.84%)	4 (28.57%)			
3	3 (7.90%)	3 (21.40%)			
Antiviral treatment time [month, median (Q1, Q3)]	60.50 (44.00, 89.50)	65.50 (40.75, 88.25)		-0.186	0.853
Consolidation treatment time [month, median (Q1, Q3)]	40.50 (36.00, 53.25)	43.00 (36.00, 56.00)		-0.052	0.958
HBsAg (log_10_ IU/mL)	3.01 (2.87, 3.46)	3.45 (3.15, 3.65)		-1.795	0.073
HBsAg ≤ 100 IU/mL	4 (10.53%)	0		0.458	0.498
AFP (ng/mL)	2.35 (1.68, 3.43)	2.00 (1.85, 2.90)		-0.310	0.757
ALT (U/L)	22.30 (13.10, 34.05)	17.60 (10.67, 25.83)		-0.990	0.322
AST (U/L)	20.40 (17.48, 26.83)	18.65 (16.30, 21.75)		-1.207	0.227
TBIL (umol/L)	10.20 (8.35, 14.35)	10.85 (8.30, 14.40)		-0.165	0.869
DBIL (umol/L)	3.70 (3.08, 4.83)	3.75 (3.18, 4.28)		-0.124	0.901
ALB (g/L)	48.25 (46.15, 50.25)	47.00 (44.18, 48.10)		-1.888	0.059
APRI [median (Q1, Q3)]	0.27 (0.20, 0.50)	0.22 (0.17, 0.33)		-1.485	0.137
APRI<1 (n, %)	36 (94.74%)	14 (100.00%)			1^*^
FIB-4 [median (Q1, Q3)]	0.85 (0.69, 1.27)	0.85 (0.55, 1.18)		-1.093	0.274
FIB-4<1.45 (n, %)	32 (84.21%)	14 (100.00%)		1.191	0.275

*Fisher’s exact test.

### Correlation between recurrence time and baseline indicators

In order to determine the relationship between the recurrence time and the antiviral treatment time, consolidation treatment time, HBsAg, ALT in CHB patients after stopping oral antiviral treatment, Spearman correlation analysis was conducted among the above indicators, virological relapse time, and clinical relapse time. The results showed that the Spearman correlation coefficients between the viral relapse and the above indicators were as, antiviral treatment time (*rs*=0.040, *P*=0.780), consolidation treatment time (*rs*=0.243, *P*=0.082), HBsAg (*rs*=-0.173, *P*=0.221), ALT (*rs*=0.081, *P*=0.568). The Spearman correlation coefficients between the clinical relapse time and the above indicators were as, antiviral treatment time (*rs*=-0.146, *P*=0.620), consolidation treatment time (*rs*=-0.149, *P*=0.612), HBsAg (*rs*=0.103, *P*=0.725), ALT (*rs*=0.437, *P*=0.119), and there was no significant correlation (*P*>0.05) (**Table 4**).

**Table 4:** Correlation between recurrence time and baseline indicators.

Variable	Viral relapse time (month)		Clinical relapse (month)	
	*rs*	*P*	*rs*	*P*
Antiviral treatment time (month)	0.040	0.780	-0.146	0.620
Consolidation treatment time (month)	0.243	0.082	-0.149	0.612
HBsAg (log_10_ IU/mL)	-0.173	0.221	0.103	0.725
ALT (U/L)	0.081	0.568	0.437	0.119

## Discussion

CHB is liver inflammation caused by HBV (3), and is one of the most important infectious diseases in China. The occurrence of CHB is the result of the interaction between the virus and its stimulated immune system. Antiviral therapy is always used to reduce the incidence of liver cirrhosis and hepatocellular carcinoma in CHB patients (17–19). Controlling HBV replication and improving human immunization are important ways to reduce liver inflammation and prevent disease progression. China’s 2019 Guidelines for the Prevention and Treatment of Chronic Hepatitis B further expanded the indications for antiviral treatment of chronic HBV infection (3). For patients with chronic HBV infection, the current major antiviral treatment are NA and interferon, both of which have advantages and disadvantages (3). The clinical cure rate of interferon is higher than that of NA, which can significantly reduce the risk of liver cirrhosis and hepatocellular carcinoma (20, 21). However, it is expensive and is inconvenient to use. Some patients stop interferon treatment because they cannot tolerate side effects. NA is easy to use and can effectively suppressed virus replication to delay the progress of liver disease, but they often relapse after drug withdrawal and need long-term treatment. Compared with interferon, NA have no direct immunomodulatory effect (3, 4, 7). Therefore, the limited course of NA cannot induce a lasting response after drug withdrawal theoretically. However, in recent years, some scholars have proposed that if patients with CHB can maintain virological response and consolidate treatment for a certain period of time, they can also consider discontinuing the drug even if HBsAg don’t turn negative (22, 23). Some studies have shown that the negative rate of HBsAg after drug withdrawal is higher than that during treatment (22). At present, domestic and foreign guidelines have proposed that it is feasible to stop oral antiviral treatment for patients with long-term oral NA before HBsAg turns negative (3, 4, 7, 24). However, the sustained virological response rate and safety after drug withdrawal still need to be verified. At present, the research on patients with liver cirrhosis after drug withdrawal is limited. According to the existing studies, patients with liver cirrhosis are not recommended to stop oral antiviral treatment. Therefore, we didn’t include patients with liver cirrhosis in this study. The purpose of our study is to establish a follow-up cohort of non-liver cirrhosis HBeAg positive initially CHB patients who stopped taking NA, observe the sustained virological response and relapse and explore the relevant factors, which could help explore a clinically feasible NA mode for HBeAg positive CHB patients to stop taking NA, achieve the rationalization and scientization of drug use, and obtain the maximum health economic benefits.

In this study, all enrolled patients used NA before stopping treatment, and 43.90% (36/82) of patients used two or more NAs due to drug resistance or other reasons. There is no significant statistical difference in the types of oral antiviral drugs between patients with sustained virological response and those with viral relapse. Considering that on the one hand, the sample size may not be large enough, on the other hand, patients all used first-line antiviral drugs such as ETV and TDF before stopping oral antiviral treatment, and long-term ETV or TDF treatment may affect the immune response of the body, narrowing the differences between groups. The third reason is that we didn’t acquire test results before starting antiviral treatment. It is difficult to define the changes of patients’ indicators during antiviral treatment. This requires us to expand the sample in the future.

NA mainly inhibits viral replication and delay disease progression by competitively inhibiting HBV DNA polymerase, but has no significant impact on the level of covalently closed circular DNA (cccDNA), integrated HBV DNA, HBeAg, and HBsAg in the liver (3, 25–27). During the follow-up period after drug withdrawal, only 36.59% (30/82) of patients had sustained virological response, 63.41% (52/82) had viral relapse, and 17.07% (14/82) had clinical relapse. All patients have consolidated treatment for more than 3 years after achieving HBeAg seroconversion according to the guidelines, however, most patients had viral relapse during the follow-up, indicating that for HBeAg positive patients, the drug should not be stopped easily even if HBeAg seroconversion has been achieved through NA treatment. In previous studies, most of the them set viral relapse (HBV DNA>2000 IU/mL) or clinical relapse (HBV DNA>2000 IU/mL, ALT>2 × ULN) as the main evaluation index. Some studies have shown that the cumulative viral relapse rate of HBeAg positive CHB patients was 50% after stopping antiviral treatment for 2 years (28). HBV DNA is the most direct marker of HBV replication and activity with high sensitivity and specificity. Positive HBV DNA indicates HBV reactivation. For patients who have achieved HBeAg seroconversion and HBV DNA clearance after oral drug treatment, positive HBV DNA indicates that the balance between human immunity and HBV is broken, and the risk of disease progression is increased. Therefore, it is meaningful and feasible to list positive HBV DNA as the evaluation index of research. In this study, we compared the relevant factors of patients with sustained virological response and patients with viral relapse, and found no reliable factors that can predict the relapse of the virus. At present, we need to further explore other indicators to guide the safe drug withdrawal in CHB patients.

A number of studies have shown that the low concentration of HBsAg at the time of stopping oral antiviral treatment is related to the high clinical cure and the low viral relapse and clinical relapse after stopping antiviral treatment (29–32). Our research supports this view. In this study, patients with sustained virological response had lower HBsAg levels than those with viral relapse. At the same time, the HBsAg level in patients with viral relapse was also lower than that in patients with clinical relapse, suggesting that the HBsAg level may be a reliable marker for evaluating the sustained virological response after drug withdrawal. HBsAg level in serum is in direct proportion to the cccDNA and integrated HBV DNA in liver tissue (27). When the host is infected with HBV, HBV enters the liver cell through sodium taurocholate cotransporting polypeptide (NTCP) on the surface of the liver cell membrane (3, 27), and forms cccDNA in the host liver cell. On the one hand, cccDNA maintains its own level stability through transcription and reverse transcription, and on the other hand, it releases HBsAg into the blood through transcriptional and translation (3, 27). Therefore, HBsAg level in serum can partly reflect the transcription activity of cccDNA *in vivo*. The lower level of HBsAg, the lower content and transcription of cccDNA in patients. At present, there is no drug that can help the host clear the cccDNA in the liver, which is the main reason why CHB is easy to relapse after drug withdrawal. In this study, HBsAg level decreased slightly after 6 months, while the viral relapse group increased slightly. However, there was no significant difference in the HBsAg level between the two groups. On the one hand, the sample size of this study is relatively small, and on the other hand, it is related to the relatively short follow-up time.

Previous research shows that serum HBcAb level can also reflect liver inflammation and predict the efficacy of antiviral therapy (33). A prospective study involving 100 patients showed that 21% of patients with HBcAb ≥ 1000 IU/mL at the end of treatment had clinical relapse within 4 years after discontinuing antiviral therapy, while 85% of patients with HBcAb < 100 IU/mL had clinical relapse. The study showed that HBcAb level was related to the clinical relapse after drug withdrawal, and it might guide CHB patients stop antiviral treatment safely (34). Unfortunately, in this study, we did not conduct quantitative analysis of HBcAb, which needs to be perfected in future studies.

In this study, 10 patients with clinical relapse received NA treatment again, and 4 patients received combination treatment of NA and peg-interferon α-2a, 1 case achieved clinical cure, and no adverse events such as cirrhosis or hepatocellular carcinoma occurred during the follow-up. Although only one case achieved clinical cure after combination treatment, it also provided a new direction for the clinical treatment of such patients. The outcome of CHB is related to the interaction between HBV and host immunity. Long term low-level antigen stimulation and long-term use of NA will affect the function of HBV specific CD8 cells (35). Studies have shown that the negative change of HBsAg after drug withdrawal was higher than that during treatment, and the negative rate of HBsAg in patients without relapse after drug withdrawal was the highest, followed by patients with relapse and untreated (22). In this study, no patient’s HBsAg converted to negative within 1 year after stopping oral antiviral treatment, 3 patients had HBsAg decrease > 1 log_10_IU/mL, and 1 patient achieved clinical cure through combined treatment after clinical relapse. It is suggested that for patients with clinical relapse after drug withdrawal, the use of peg-interferon α-2a may be helpful to achieve clinical cure (HBsAg turns negative). The disappearance of HBsAg is considered as “clinical cure”, which is an ideal treatment target for the antiviral treatment of chronic hepatitis B infection (36). In order to achieve the immune control goal characterized by HBeAg seroconversion or HBsAg disappearance, interferon has greater advantages than NA. Because interferon treatment has higher HBeAg seroconversion rate and HBsAg disappearance rate than NA, and once interferon treatment achieves HBeAg seroconversion or HBsAg disappearance, it will bring long-term good clinical outcomes after stopping treatment (37).

In conclusion, our study shows that according to the existing drug withdrawal criteria, the rate of sustained off-treatment virological response of CHB patients with HBeAg positive initially is low, and it always occurred in patients with low HBsAg levels. Patients are more likely to have viral relapse or clinical relapse after stopping oral antiviral treatment. The patients with clinical relapse are expected to achieve clinical cure after combining peg-interferon α-2a with NA. Due to the limited number of patients who can reach the withdrawal standard of oral antiviral drugs in clinical practice, our sample size is not large. If we can expand the sample size in future studies, the conclusion may be more reliable. And monitoring for only 12 months may overlook the incidence of viral relapse, cirrhosis and hepatocellular carcinoma, so it’s recommended to follow up at least 24 months in future studies.

## Data availability statement

The raw data supporting the conclusions of this article will be made available by the authors, without undue reservation.

## Ethics statement

The studies involving human participants were reviewed and approved by the Ethics Committee of Beijing Ditan Hospital affiliated to Capital Medical University (Jing Di Lun Ke Zi 2017 No. 003 - 02). The patients/participants provided their written informed consent to participate in this study.

## Author contributions

ML, JD, and YX contributed to the study design. ML, FS, ZL, and LH contributed to the data analysis. ML, FS, ZL, LH, WD, TJ, SYW, YL, LZ, GS, RL, SLW, MC and ML contributed to the recruitment, enrolment, and assessment of participants, as well as data collection. FS, WD, TJ, XB, HL, YG, HH, MX, and XC contributed to following up with the patients. ZZ, YJL, and LY managed all aspects of laboratory support. FS wrote the first draft of the manuscript. YX revised the manuscript and is the guarantor of the article. All authors contributed to the article and approved the submitted version.

## Funding

Project supported by The Digestive Medical Coordinated Development Center of Beijing Hospitals Authority (XXZ0302 and XXT28); Beijing science and technology commission (Z211100002921059); National Science and Technology Major Project of China (2017ZX10201201-001-006, 2017ZX10201201-002-006, 2018ZX10715-005-003-005); Beijing Hospitals Authority Clinical medicine Development of special funding support ( XMLX 202127); National Key R&D Program of China (2022YFC2603505); High-level Public Health Technical Personnel Training Program of Beijing Municipal Health Commission (2022-3-050); The capital health research and development of special (2022-1-2172).

## Conflict of interest

The authors declare that the research was conducted in the absence of any commercial or financial relationships that could be construed as a potential conflict of interest.

The reviewer YL declared a shared parent affiliation with the authors to the handling editor at the time of review.

## Publisher’s note

All claims expressed in this article are solely those of the authors and do not necessarily represent those of their affiliated organizations, or those of the publisher, the editors and the reviewers. Any product that may be evaluated in this article, or claim that may be made by its manufacturer, is not guaranteed or endorsed by the publisher.
